# Trends in social inequality and how mental wellbeing vary and covary among Norwegian adolescents and their families: the Young-HUNT Study

**DOI:** 10.1177/14034948231172634

**Published:** 2023-09-30

**Authors:** Bodil Elisabeth Valstad Aasan, Monica Lillefjell, Steinar Krokstad, Erik R. Sund

**Affiliations:** 1 HUNT Research Centre, Department of Public Health and Nursing, Faculty of Medicine and Health Sciences, Norwegian University of Science and Technology, NTNU, Levanger, Norway; 2 Levanger Hospital, Nord-Trøndelag Hospital Trust, Levanger, Norway; 3 Department of Neuromedicine and Movement Science, Faculty of Medicine and Health Sciences, Norwegian University of Science and Technology, Trondheim, Norway; 4 Department of Public Health, Faculty of Health Sciences, University of Stavanger, Stavanger, Norway; 5 Faculty of Nursing and Health Sciences, Nord University, Levanger, Norway

**Keywords:** Adolescents, education level, loneliness, multivariate multilevel model, psychological distress, social inequality, socioeconomic, Young-HUNT Study

## Abstract

**Background:**

The study had two aims: first, to investigate trends in socioeconomic inequalities in psychological distress and loneliness among Norwegian adolescents, and second, to study variation and covariation of psychological distress and loneliness within adolescents and between siblings within families.

**Methods:**

Multivariate mixed models were used to investigate trends in socioeconomic inequality in psychological distress and loneliness using three separate cohorts of Norwegian adolescents from the Young-HUNT study conducted in 1995–1997 (Young-HUNT1, *n* = 8980), 2006–2008 (Young-HUNT3, *n* = 8199) and 2017–2019 (Young-HUNT4, *n* = 8066). Register data on parental education level was used as a marker of socioeconomic position (SEP), and a unique family number was used to identify adolescents belonging to the same family. A three-level multivariate mixed model was created, consisting of the outcomes at level 1, adolescents at level 2 and families at level 3.

**Results:**

No statistically significant difference in scores on loneliness and psychological distress was observed between low and high parental education level in Young-HUNT1, whereas in Young-HUNT4, low parental education level was associated with a higher score on both psychological distress (β = 0.09; 95% confidence interval (CI), 0.03–0.14) and loneliness (β = 0.12; 95% CI 0.07–0.17). Analyses of covariation between psychological distress and loneliness showed that they were correlated within adolescents and strongly correlated within families across all timepoints.

**Conclusions:**

**Increasing socioeconomic inequalities in psychological distress and loneliness among Norwegian adolescents is worrisome. Further, the family seems to be an important arena for potential prevention of psychological distress and loneliness among adolescents, regardless of parental education level**.

## Introduction

Since the early 2010s, mental health problems and loneliness have increased sharply among adolescents in Western societies, especially among girls [1,2]. It is well-established that mental health problems and loneliness are associated, and studies indicate that they influence each other reciprocally. Longitudinal studies have shown that loneliness increases the risk of becoming depressed [3], and may worsen depressive symptoms among individuals who are already depressed [4]. Additionally, social withdrawal is common among both depressed and anxious children and adolescents, which may cause or reinforce loneliness [5].

Socioeconomic position (SEP) has been found to be an important determinant of health and wellbeing across the life course [6], and several theories have been proposed as explanations of how socioeconomic inequalities in health arise. Social selection theories suggest that people of lower SEP may possess certain personal characteristics (e.g., risk of poor health), which limits their ability to move upward on the socioeconomic ladder [7]. Social causation theories highlight how the conditions in which people live and grow are generally worse among people of lower SEP, which increases their risk of poor health and wellbeing [6,7]. For example, differences in material, psychosocial and behavioural factors have been proposed as important mechanisms of socioeconomic inequalities in health among the adult and adolescent population [8,9].

Studies investigating trends in socioeconomic inequalities in adolescent mental health and loneliness have produced somewhat inconsistent results. One study found stable socioeconomic inequalities in adolescents subjective health complaints between 1994 and 2010 [10], whereas another study reported increased socioeconomic inequalities in psychological symptoms between 2002 and 2010 [11]. Further, a study from Denmark showed decreased socioeconomic inequalities in loneliness due to a sharper increase in loneliness among adolescents from higher SEP compared with lower SEP [12].

Mitigating socioeconomic inequalities in mental health and loneliness among children and adolescents is important, and the family context may be essential for understanding and mitigating these inequalities as the family’s SEP describes their level of access to, and control over, economic and social resources relative to other families. Moreover, family SEP may impact the mental health and loneliness of children and adolescents in several ways. The Family Stress Model (FSM) postulates that economic hardship may lead to parental psychological distress and disrupted parenting, which could affect the mental health and adjustment of their children [13]. Further, higher education is associated with health literacy, the ability to process and utilize health information, which could be important for parents to help and support their children effectively [14]. Additionally, psychosocial factors such as social ranking may impact the mental health and loneliness of children and adolescents as children and adolescents compare their families with others [15]. Consequently, it is important to situate adolescents within the wider family context when investigating socioeconomic inequalities in health. Such information could be important for developing effective interventions for mitigating socioeconomic inequalities.

### Aims

The present study consists of two main investigations using multivariate mixed models. First, we investigate trends in socioeconomic inequalities in psychological distress and loneliness using three separate cohorts of adolescents 11 years apart spanning the years 1995 to 2019. Second, we examine to what extent psychological distress and loneliness vary and covary within and between individuals and families over time and to what extent variation between families can be explained by parental education level.

## Methods

### Study population

Our study includes adolescents between 13 and 19 years of age from three separate cohort waves of the Young-HUNT study in Norway [16]. The total youth population residing in the northern part of the county Trøndelag (formerly known as Nord-Trøndelag) was invited to the surveys. The county consists of rural and small urban areas, and the population is both ethnically and socioeconomically homogeneous. The county is fairly representative of Norway in terms of geography and demography [16]. The three cross-sectional surveys were conducted in 1995–1997 (Young-HUNT1), 2006–2008 (Young-HUNT3) and 2017–2019 (Young-HUNT4), consisting of 8980, 8199 and 8066 participants, respectively. Response rates were 88%, 78% and 76%, respectively. Data were linked through a unique person identifier (a Norwegian personal ID number) to registry data from Statistics Norway containing information on family IDs and parental education level. All participants gave their informed written consent. For adolescents younger than 16 years, their parents provided written consent. The current study was approved by the Regional Committee for Medical and Health Research Ethics (REK-Midt ref. 262408).

### Psychological distress

Psychological distress was assessed using the Symptom Checklist 5 (SCL-5) [17], which measures symptoms of depression and anxiety. The questionnaire is a shortened version of the Hopkins Symptom Checklist (HSCL) [17]. The adolescents were asked to report if they had experienced the following statements during the last 14 days: ‘Been constantly afraid and anxious’; ‘Felt tense or uneasy’; ‘Felt hopelessness when you think of the future’; ‘Felt dejected or sad’; ‘Worried too much about various things’. The items were answered on a four-point scale from ‘not bothered’ to ‘very much bothered’, resulting in a mean score from one to four, in which a higher score indicated a higher symptom burden.

### Loneliness

Loneliness was assessed using one questionnaire item. In Young-HUNT1 and Young-HUNT3, the participants were asked ‘Do you often feel lonely?’, answered on a five-point scale from ‘very often’ to ‘very seldom or never’. In Young-HUNT4, the participants were asked: ‘At school or during my spare time. How often do you feel that you are lonely’, answered on a five-point scale from ‘very rarely or never’ to ‘very often’. The loneliness variable was coded such that a higher score indicated a higher score of loneliness.

### Parental education level

Data on parental education level were used as a measure of SEP and were obtained from Statistics Norway for both parents. Based on a family ID number from Statistics Norway, we formed a parental education variable defined as low or high. High refers to families where at least one parent had received a higher education at the university level. The family ID was also used to link adolescents and parents to form families.

### Statistical analyses

**Figure 1.**
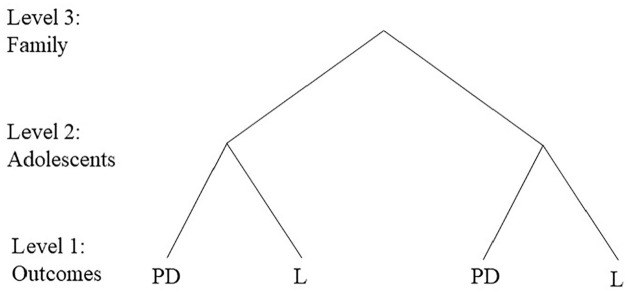
Multivariate multilevel structure of the outcomes psychological distress and loneliness (level 1), nested within adolescents (level 2), nested within families (level 3). L, loneliness; PD, psychological distress.

Multivariate linear mixed models were used to investigate trends in socioeconomic inequality and how psychological distress and loneliness vary and covary within and between adolescents and their families using three levels. Such a model makes it possible to investigate the degree of variance between adolescents and between families, as well as whether levels of psychological distress and loneliness correlate within adolescents and within families. The outcome measures, psychological distress and loneliness, constituted the lowest level in the model. The lowest level exists solely to define the multivariate structure and no variation was specified [18]. The two outcomes were nested within adolescents at level 2, who were, in turn, nested within their families at level 3. See Figure 1 for an illustration of the hierarchical model. The statistical framework allowed us, first, to examine whether the asso-ciation between parental education and outcome differed by outcome type (psychological distress versus loneliness). This enabled us to compare the fixed effects across outcomes. Second, the random part of the models allowed us to examine the extent to which the outcomes vary and covary within and between individuals and families. The level 2 variances and covariances represent the between-individual variances and covariances; similarly, the level 3 variances and covariances describes the between-family variances and covariances. The between-family variances and covariances are based on the average score on psychological distress and the average score on loneliness between siblings within families. The analyses were conducted separately for each of the three surveys (Young-HUNT1, Young-HUNT3 and Young-HU-NT4). The outcomes, both standardised to have a mean of 0 and a standard deviation of 1, were treated as continuous variables, and the reported models for each survey included age (centered at age 16 in all surveys), sex and parental education as covariates. We excluded 200 (2.2%) participants from Young-HUN-T1, 204 (2.5%) participants from Young-HUNT3 and 364 (4.5%) participants from Young-HUNT4 due to missing parental education or family identifier from Statistics Norway. Mann-Whitney *U*-tests were used to test the difference in mean scores on the outcomes between surveys. Data management, descriptive statistics and the Mann-Whitney *U*-tests were conducted using Stata version 17. The multivariate mixed analyses were performed in MLwiN version 3.05 [19], and we report beta coefficients with 95% confidence intervals (CI) for the fixed effect estimates, and variances, covariances and standard error (SE) for the random effect estimates.

## Results

### Descriptive statistics

A total of 25,245 (50.3% girls) adolescents were included in the present study (Table I). The distribution of boys and girls and the mean age were similar across the three surveys. Results from the Mann-Whitney *U*-tests showed a statistically significant difference in mean scores on psychological distress and loneliness between Young-HUNT1 and Young-HUNT3 (psychological distress: z −3.71, *P* < 0.001; loneliness z −4.29, *P* < 0.001), and between Young-HUNT3 and Young-HUNT4 (psychological distress z −15.99, *P* < 0.001; loneliness 20.55, *P* < 0.001). Additionally, the number of participants with a high level of parental education increased from 32.6% in Young-HUNT1 to 60.1% in Young-HUNT4, following the general trend of increasing levels of education in Norwegian society [20].

**Table I.** Descriptive statistics (*N* = 25,245) for the three Young-HUNT studies.

	Young-HUNT1 (1995–1997, *n* = 8980)	Young-HUNT3 (2006–2008, *n* = 8199)	Young-HUNT4 (2017–2019, *n* = 8066)
	Mean or no. (SD or %)	Mean or no. (SD or %)	Mean or no. (SD or %)
Number of families	7099	6518	6174
Mean number of siblings	1.26	1.26	1.31
Sex			
Girls	4464 (49.7%)	4127 (50.3%)	4106 (50.9%)
Boys	4516 (50.4%)	4072 (49.7%)	3960 (49.1%)
Age	16.16 (1.84)	15.93 (1.77)	16.15 (1.83)
Parental education level			
Low	5926 (67.4%)	4354 (54.4%)	3075 (39.9%)
High	2863 (32.6%)	3648 (45.6%)	4629 (60.1%)
Missing	200 (2.2%)	204 (2.5%)	364 (4.5%)
Outcomes			
Psychological distress	1.45 (0.49)	1.50 (0.55)	1.70 (0.72)
Loneliness	2.04 (0.94)	2.14 (1.07)	1.87 (1.15)

SD, standard deviation.

### Trends in associations between age and sex and psychological distress and loneliness

Across the three surveys, higher age was associated with a higher score on psychological distress and loneliness, and the strength of the association increased across surveys (Table II).

**Table II.** Fixed and random effect estimates for psychological distress and loneliness for adolescents in the Young-HUNT studies.^a^

	Young-HUNT1 (1995-97)			Young-HUNT3 (2006-08)			Young-HUNT4 (2017-19)		
	Estimate	95%	CI	Estimate	95%	CI	Estimate	95%	CI
Fixed effects									
Psychological distress									
Age	0.07	0.06	0.08	0.08	0.07	0.09	0.10	0.09	0.11
Sex									
Boys	0	reference		0	reference		0	reference	
Girls	0.37	0.34	0.40	0.50	0.46	0.54	0.85	0.80	0.90
Parental education									
High Education	0	reference		0	reference		0	reference	
Low Education	−0.03	−0.07	0.01	−0.01	−0.05	0.04	0.09	0.03	0.14
Loneliness									
Age	0.03	0.02	0.04	0.05	0.03	0.06	0.07	0.05	0.08
Sex									
Boys	0	reference		0	reference		0	reference	
Girls	0.24	0.20	0.28	0.35	0.31	0.40	0.41	0.36	0.46
Parental education									
High Education	0	reference		0	reference		0	reference	
Low Education	−0.01	−0.05	0.03	0.06	0.02	0.11	0.12	0.07	0.17
Random effects									
Between-family variance									
Variance PD (SE)	0.086	(0.015)		0.100	(0.020)		0.217	(0.032)	
Variance L (SE)	0.079	(0.019)		0.089	(0.027)		0.146	(0.030)	
Covariance PD/L (SE)	0.067	(0.013)		0.086	(0.019)		0.163	(0.025)	
Correlation PD/L	0.81			0.91			0.91		
Between-individual variance									
Variance PD (SE)	0.533	(0.017)		0.660	(0.022)		0.992	(0.034)	
Variance L (SE)	0.692	(0.021)		0.886	(0.030)		0.971	(0.032)	
Covariance PD/L (SE)	0.258	(0.014)		0.391	(0.020)		0.475	(0.026)	
Correlation PD/L	0.42			0.51			0.48		

a Estimates (beta coefficients) and 95% CI from the fixed part of a multivariate multilevel of psychological distress and loneliness. Random effects display variation and covariation (with SE) in psychological distress and loneliness between families and adolescents.

CI, confidence interval; L, loneliness; PD, psychological distress; SE, standard error.

In all three surveys, girls showed a higher average of psychological distress and loneliness than boys. The strength of the association increased from Young-HUNT1 to Young-HUNT4. Compared with boys, girls scored a 0.37 SD higher (β = 0.37; 95% CI 0.34–0.40) on psychological distress in Young-HUNT1 and the difference increased to 0.85 in Young-HUNT4 (β = 0.85; 95% CI 0.80–0.90).

### Trends in socioeconomic inequalities in psychological distress and loneliness

Results of the analyses of socioeconomic inequality in psychological distress showed no statistically significant difference between adolescents of a low or high parental education in Young-HUNT1 and Young-HUNT3. However, adolescents from low educated parents had a higher score of psychological distress (β = 0.09; 95% CI 0.03–0.14) in Young-HUNT4.

For loneliness, there was no statistically significant difference between high and low parental education in Young-HUNT1. In Young-HUNT3 and Young-HUNT4, a lower parental education level was significantly associated with a higher score on loneliness (β = 0.06; 95% CI 0.02–0.11 and β = 0.12; 95% CI 0.07–0.17, respectively).

### Variation and covariation within and between individuals and families

The random effects displayed at the bottom half of Table II show how psychological distress and loneliness varied across individuals and families in each Young-HUNT survey, as well as their correlation at both levels. In Young-HUNT1, the within-individual correlation between psychological distress and loneliness was 0.42—a figure that increased to 0.51 in Young-HUNT3, before decreasing slightly to 0.48 in Young-HUNT4. The within-family correlation between psychological distress and loneliness (i.e. how the average score on psychological distress among siblings within families correlates with the average score on loneliness) showed an increase from 0.81 in Young-HUNT1 to 0.91 in Young-HUNT4. The proportion of the variance located at the family level (family-level variance/family-level variance + individual-level variance) was relatively stable for both outcomes in Young-HUNT1 (psychological distress, 0.14; loneliness, 0.10) and Young-HUNT3 (psychological distress, 0.13; loneliness, 0.09) but increased in Young-HUNT4 (psychological distress, 0.18; loneliness, 0.13). Lastly, the between-family variance was not explained by parental education in any of the Young-HUNT surveys.

## Discussion

The present results suggest an increase in socioeconomic inequalities in psychological distress and loneliness among adolescents during the period from 1995 to 2019. No statistically significant difference between low and high parental education level was found for either psychological distress or loneliness in Young-HUNT1. In Young-HUNT3, a low parental education level was associated with higher levels of loneliness, while in Young-HUNT4, a low parental education level was associated with both higher loneliness and psychological distress.

Findings from the models’ random effects showed that the between-individual variability and the between-family variability remained stable across the three surveys, and that the between-family variance was not explained by parental education. Further, both within adolescents and their families, psychological distress and loneliness were positively correlated. A particularly high correlation between outcomes was observed within families across all surveys.

The present findings align with the results of Elgar et al [11], which showed increased socioeconomic inequalities in psychological symptoms among adolescents in 34 countries between 2002 and 2010. However, a recent study of Norwegian adolescents reported stable socioeconomic inequalities in psychological distress and loneliness between 2014 and 2018 [21], while decreased socioeconomic inequality in loneliness was observed among Danish adolescents between 1991 to 2014 [12]. Discrepancies in results could be due to how SEP was measured, differences in country context and the time span of the analyses.

However, various explanations for the persistence of socioeconomic inequalities have been put forward [22]. For example, continued socioeconomic inequalities in health may indicate that the welfare state has failed to mitigate material and immaterial inequalities [22]. Furthermore, others have proposed that the group of people with low education has become more homogenous through intergenerational mobility [22]. Hence, the inequalities reported may be a methodological artefact due to differences in the composition of the groups over time.

Additionally, the social significance of education may have changed as well [23]. On the one hand, completing higher education may become less socially significant as such an achievement becomes more common. On the other hand, this intergenerational mobility over time could have negative psychosocial consequences for those who do not complete higher education. Social rank theory postulates that people socially rank themselves with others, and may be a potential psychosocial mechanism of socioeconomic inequalities in mental health as people who consider themselves of lower rank compared with their peers have worse mental health [15]. Thus, as the proportion of low-educated people is becoming smaller and potentially more marginalized, this may have a negative impact on their mental health through psychosocial factors such as social ranking. Further, the current use of social media among adolescents is likely to amplify these social comparison effects [24].

Regarding the between-family variance, we did not find any evidence that parental education level explained any between-family variance for either outcome. This finding may be expected based on the non-existent or weak effect sizes for parental education in the present study. However, in accordance with previous studies [25], a higher score on psychological distress was associated with a higher score on loneliness within adolescents and especially within families. The high within-family correlation may not be surprising as siblings often share genetics and environment, and studies have found that siblings often experience similar symptomology [26]. Thus, the present results may suggest that the family context is an important arena for preventing mental health problems and loneliness among adolescents. For example, interventions may aim to improve potentially shared familial environmental factors known to be associated with the mental health and loneliness of adolescents, such as parenting practices, family stress, interparental conflict, and parental mental health problems [13,27,28]. Thus, family intervention programs may be important for preventing mental health problems and loneliness in children and adolescents as improvement in the family environment may benefit all siblings within a given family.

Though we observed weak effect sizes, there seems to be an emerging group of adolescents from low parental education who experiences higher levels of psychological distress and loneliness compared with high parental education, suggesting that interventions aimed at this group may be needed. Considering the importance of the family context, family programs may be important in reducing psychological distress and loneliness among all adolescents, as well as mitigating socioeconomic inequalities. For example, some family programs combines a universal and selective intervention approach by providing appropriate levels of support for what the family seeks and needs using the principle of proportionate universalism [29].

Though previous research has highlighted the importance of material, psychosocial and behavioural factors in explaining socioeconomic inequalities among adolescents [9], it is important to consider how conditions in which we live and grow have changed. For example, does the current use of social media affect adolescents from various social groups differently? Such information could be important for developing effective interventions to mitigate socioeconomic inequalities in psychological distress and loneliness among adolescents.

### Strengths and limitations

The present study has several strengths. Firstly, the study includes a large sample of adolescents from the general population. Secondly, the participation rate was high at all three survey points, and there were few missing values. Thirdly, compared with previous trend studies of social inequality, the multilevel modelling approach adds information about the relative importance of individual factors and family factors as well as the covariation of psychological distress and loneliness across the time-points. However, some limitations of the study must be mentioned. The sample population was derived from a county in mid-Norway, which is somewhat more egalitarian than more urban populations within Norway. Thus, the socioeconomic inequalities presented here are likely a conservative estimate. Further, SEP was measured using only parental education level, which could present a limited measure of SEP. Additionally, psychological distress was measured by five items and loneliness by one item, which may decrease the accuracy of the measurement of the outcomes.

## Conclusion

The results of the present study indicate that there has been an increase in social inequality in psychological distress and loneliness among adolescents in Norway. Further, the level of psychological distress was associated with the levels of loneliness within the adolescents and within their families. It is important to continue investigating trends in social inequality related to health. The degree of similarity in psychological distress and loneliness between siblings within families suggest that family intervention programs should be considered in public health work.
